# Overexpression of cathepsin f, matrix metalloproteinases 11 and 12 in cervical cancer

**DOI:** 10.1186/1471-2407-5-68

**Published:** 2005-06-30

**Authors:** Guelaguetza Vazquez-Ortiz, Patricia Pina-Sanchez, Karla Vazquez, Alfonso Duenas, Lucia Taja, Patricia Mendoza, José A Garcia, Mauricio Salcedo

**Affiliations:** 1 Oncogenomics Laboratory, Oncology Research Unit, Oncology Hospital, National Medical Center SXXI-IMSS, Mexico; 2 Division of Basic Research, INCAN, SS, Mexico; 3 Laboratory of Theoretical Biology, Research Department, La Salle University, Mexico

## Abstract

**Background:**

Cervical carcinoma (CC) is one of the most common cancers among women worldwide and the first cause of death among the Mexican female population. CC progression shows a continuum of neoplastic transitions until invasion. Matrix metalloproteinases (MMPs) and cathepsins play a central role on the enhancement of tumor-induced angiogenesis, cell migration, proliferation, apoptosis and connective tissue degradation. MMPs -2 and -9 expression has been widely studied in cervical cancer. Nevertheless, no other metalloproteinases or cathepsins have been yet related with the progression and/or invasion of this type of cancer.

**Methods:**

Three HPV18 CC cell lines, two HPV16 CC cell lines and three HPV16 tumor CC tissues were compared with three morphologically normal, HPV negative, cervical specimens by cDNA arrays. Overexpression of selected genes was confirmed by end point semiquantitative reverse transcription-PCR with densitometry. *In situ *hybridization and protein expression of selected genes was further studied by means of two tissue microarrays, one consisting of 10 HSIL and 15 CC and the other one of 15 normal cervical and 10 LSIL tissues.

**Results:**

*TIMP1, Integrins alpha 1 and 4, cadherin 2 and 11, Cathepsins F, B L2, MMP 9, 10 11 and 12 *were upregulated and *Cathepsin S, L, H and C, Cadherins 3 *and *4, TIMP3, MMP 13, Elastase 2 *and *Integrin beta 8 *were found to be downregulated by cDNA arrays. Endpoint RT-PCR with densitometry gave consistent results with the cDNA array findings for all three genes selected for study (*CTSF, MMP11 *and *MMP12). In situ *hybridization of all three genes confirmed overexpression in all the HSIL and CC. Two of the selected proteins were detected in LSIL, HSIL and CC by immunohistochemistry.

**Conclusion:**

Novel undetected CC promoting genes have been identified. Increased transcription of these genes may result in overexpression of proteins, such as CTSF, MMP11 and MMP12 which could contribute to the pathogenesis of CC.

## Background

Cervical carcinoma (CC) is one of the most common cancers among women worldwide and the first cause of death among the Mexican female population[1]. High-risk human papillomavirus (HPV) infection is considered the most important risk factor associated with the development of this tumor, and it is present in the 99.7% of invasive cervical tumors worldwide[2]. CC progression shows a continuum of neoplastic transitions from low grade squamous intraepithelial lesion (LSIL), High grade squamous intraepithelial lesion (HSIL), to invasive cervical cancer. Tumor invasion and metastasis are key steps in the progression of malignant tumors, which involves both extracellular matrix (ECM) and basement membrane degradation[3]. Although several classes of proteolytic enzymes are involved in these processes[4], Matrix metalloproteinases (MMPs) play a central role on the enhancement of tumor-induced angiogenesis, cell migration, proliferation, apoptosis and connective tissue degradation [5,6]. Different MMP family members have been identified, including collagenases (MMP-1, -8 and -13), gelatinases (MMP-2 and -9), stromelysins (MMP-3, -10, -11), membrane associated (MMP-14, -15, -16, -17, -23, -24, -25) and other types of MMPs, such as the Metalloeslastase (MMP-12)[7]. MMPs -2 and -9 have been widely studied. Increased mRNA and protein levels of both MMP-2 and MMP-9 have been detected in breast, colon, pancreatic and cervical cancers[8].

Human macrophage metalloelastase (MMP-12) has been identified in alveolar macrophages of cigarette smokers as an elastolytic MMP[9]. It can degrade elastin, and other substrates, such as type IV collagen, fibronectin, laminin, gelatin, vitronectin, entactin, heparin, and chondroitin sulphates[10]. MMP-12 is overexpressed by macrophages in atherosclerotic lesions[11] and in intestinal ulcerations[12]. It has been demonstrated that besides macrophages, transformed epithelial cells can express MMP-12 in skin and vulvar cancers; MMP12 expression levels correlate with epithelial dedifferentiation and histological aggressiveness [13,14].

Stromelysin 3 ST3 (MMP-11) is another protease that can modulate cancer progression by remodelling extracellular matrix. It cleaves α1-antitripsin and IGF-BP1[15]. Normal ST3 expression is present during embriogenesis and wound healing, and its expression in stressed epithelial cells is detected in the vicinity of fibroblasts[16]. Malignant epithelial cells depend on local stromal cells including fibroblastic, endothelial and inflammatory cells to develop primary and secondary tumors. ST3 expression is observed in the area which surrounds malignant epithelial tumour cells and sometimes in tumor cells of esophageal, oral, papillary thyroid, colorectal, skin and ovarian carcinomas [17-22]. Hence, ST3 gene expression appears to be associated with tumor progression[23].

Cathepsins are cysteine proteases from the papain family that are responsible for protein breakdown in lysosomes. The human Cathepsin family is composed by Cathepsins B, C, F, H, K, L, O, S V, W and X[24]. The recently described Cathepsin F has been demonstrated by Northern blot analysis, to be ubiquitously expressed in several tissues with a higher expression in skeletal muscle and testis. Cathepsin F transcripts were also found in several cancer cell lines, suggesting that this enzime could be involved in degradative processes during tumor progression[25].

In the present study we examined mRNA expression levels of 8,000 genes including MMPs and cathepsins by means of cDNA microarrays in CC samples and cell lines. Selected genes were confirmed to be up-regulated by endpoint RT-PCR and densitometry. Moreover, RNA and protein expression of the selected genes were further examined by *in situ *hybridization and IHC applied on Tissue Microarrays (TMAs)[26]. Several genes that were either up- or down-regulated in CC were detected, raising the possibility that these genes could play an active or a passive role in the malignant progression of CC.

## Methods

### Cell lines and tissue samples for cDNA arrays and RT-PCR

Hela (HPV18), SiHa (HPV16) and CaSki (HPV16) cell lines were grown in the recommended media. The cell lines designated CaLO (derived from Invasive squamous carcinoma Stage IIB, HPV18) and INBL (derived from Invasive Squamous carcinoma IV A, HPV18), were established at the FES Zaragoza-UNAM, Mexico [27]. Fresh biopsies of dysplastic lesions were taken during colposcopy from 20 patients at the National Center for Dysplasias, in Mexico City. Twenty two normal cervical tissues from pre-menopausal women who died from non-gynecological related causes were obtained within the first two hours *post-mortem *at the Pathology Department of the Hospital General de Mexico, S. S. All the described procedures were evaluated and approved by the local committee of ethics of the Mexican Institute of Social Security; we also obtained written informed consent from the patients. All tissue samples were divided in three sections, the central part was snapped frozen in liquid nitrogen and stored at -70°C until nucleic acid extraction, and the extremes from the biopsies were fixed overnight in 70% ethanol and paraffin embedded at the Department of Pathology, Oncology Hospital, National Medical Center SXXI, Mexico. Serial sections from the extremes of the biopsies stained by Haematoxilin/Eosin were inspected for representativity of the tissue. Carcinoma samples were considered as representative when at least 70% of all cells in the tissue section consisted of cancer cells. Normal tissue samples were considered as such when at least 80% of the sample consisted of microscopically ectocervical normal tissue and HPV infection absence. All the invasive samples were diagnosed as squamous lesions.

### Tissue samples for ISH and IHC

Paraffin blocks of 35 patients: 10 HSIL, 15 CC, and 10 LSIL were randomly collected from the files of the National Center of Dysplasias in Mexico City. Fifteen normal cervical tissue samples (obtained *post-mortem*) were fixed in buffered formaldehyde and embedded in paraffin for preparation of the TMA blocks. The TMAs were constructed as previously described[28]. One of the TMAs contained 10 HSIL tissues and CC samples; the other was made with the 15 normal cervical tissues and 10 LSIL samples. The TMAs did not include the investigated samples by the cDNA arrays or by RT-PCR.

### Nucleic acid isolation for probe preparation and RT-PCR

Both RNA and DNA were Isolated from all tissue samples which met the required criteria: squamous cell carcinoma, HPV type 16 or 18, at least 70% cancer cells in the biopsy, and from the cervical cancer cell lines using the Trizol Reagent (Gibco BRL, Life Technologies, Grand Island NY USA) according to manufacturer's conditions. Each DNA sample was subjected to HPV typing by polymerase chain reaction (PCR) using the MY09/MY11 consensus primers and direct sequencing. Potentially contaminating DNA was removed by RNAse-free DNAse I treatment (Ambion Inc. Austin Texas USA). The resulting RNA concentration was measured spectrophotometrically and the quality of both nucleic acids was confirmed in agarose gels.

### cDNA probe preparation

cDNAs were synthesized according to Ambion Advantage System User Manual. These samples were: cell lines HeLa, SiHa, CaSki, CALO and INBL, three HPV16+ tumor samples (T07, T31, T64) and three HPV^- ^normal samples (N03, N11, N22). Unfortunately no HPV18+ tumor samples were available for array hybridization due to insufficient RNA yield for probe preparation. The cDNA probes were obtained by RT-PCR from 500 ng RNA in the presence of (α-^33^P) dATP (3000 Ci/mmol, NEN). Briefly, RNAs were denatured at 70°C for 10 min and cDNAs were synthetized at 42°C by oligo-dT priming in a final volume of 30 μl. The labelled cDNAs were purified by spin column chromatography. The final incorporation of the radiolabel was approximately 1 × 10^8 ^cpm per reaction.

### Hybridization and array analysis

ULTRArray Advantage System array blots (Ambion Inc. Austin Texas USA) containing 8400 genes were prehybridized at 68°C for at least 90 min before probe addition in hybridization buffer. Then, 1.5 × 10^7 ^cpm of each labeled cDNA was added to the buffer. Hybridization was performed at 60°C overnight in a rolling bottle. The arrays were washed twice with 2X SSC and 0.5 % SDS at 60°C for 30 min; followed by two stringent washes with 0.5X SSC, 0.5% SDS at the same temperature and for the same length of time. Finally, damp arrays were sealed in plastic wrap and exposed to imaging plates (BASMP 2040S; Fuji, Nakamura, Japan) for 24 hours, which were then scanned with a STORM 860 phosphorimager/fluorimager (Amersham Biosciences, Buckinghamshire UK) to obtain 16-bit images. ArrayVision software (Image Research Inc) was used for the analysis. The artifacts were eliminated and the intensity of each spot was analyzed after background substraction. Then, data was normalized and analyzed by using limma bioconductors and marray package [29,30] for all genes presented in the array and the means of the normal array (three normal samples) were obtained. The frequency of positive cases was obtained by comparing the mean normal array with each individual tumor or cell line array. The threshold frequency was set at 40% and the intensity ratio (tumor;normal) threshold values of 4 up-regulation and -1 for down-regulation present in all samples were used in an attempt to detect significant expression changes.

### RT-PCR analysis

Estimation of the reliability of the cDNA results for the three selected genes was performed by endpoint RT-PCR with densitometry. These samples were previously described; four normal samples, 5 cell lines, 3 HPV16 and 2 HPV18 tumors; displaying a clear change in their expression in respect to the normal, independently of the HPV type present. Two-hundred ng of total RNA from all samples were reverse transcribed using the RT-PCR Access System (Promega, Madison, WI). Primers for the genes of interest were: Cathepsin F (CTSF) 396 bp, TM = 67°C, (5'-GTGCTGATCAGAGTGGCTGCTGC-3' and 5'-AGTTTCCYGGACATGGAGAGGGAC-3'); Matrix Metalloproteinase 12 (MMP12), 370 bp TM = 55°C (5'-TCACGAGATTGGCCATTCCTT-3' and 5'-TCTGGCTTCAATTTCATAAGC-3')[31]; and Matrix Metalloproteinase 11 (MMP11/ STMY3), 399 bp, TM = 66°C, (5'-CCATGGCAGTTGGTGCAGGAGCAG-3' and 5'-TGCAGTCATCTGGGCTGAGACTCA-3'); and β-actin: (5'-TGAAGTCTGACGTGGACATC-3' and GTTCGTTCCTCATACTGCTCA-3') 243 bp, TM = 55°C primers were designed using Integrated DNA technologies Biotools software . The RT-PCR conditions were: for the first strand synthesis of cDNA 48°C for 45 min and 94°C for 2 min to denature template; and for second strand synthesis and DNA amplification: 94°C for 30 sec, (specific TM°C for each set of primers) for 1 min, and 68°C for 2 min for a total of 24 cycles, followed by a single step at 68°C for 7 min. The products were visualized on 1.5% agarose gels stained with ethidium bromide, and signals were quantified by densitometry using MetaView analysing system (version 4.5 Universal Imaging Corp., USA). *MMP11, MMP12 *and *CTS *expression was standardized to β-actin expression assessed from the same cDNA in separate PCR reactions and run in parallel on separate gels. The standardized mean of each triplicate PCR was then expressed relative to the levels in β-actin cDNA. Statistical comparisons among groups were analyzed by a Kruskall Wallis-test. A *P *value of <0.05 was considered as statistically significant.

### *In situ *hybridization (ISH)

Briefly, five-micron tissue sections were obtained from TMA paraffin blocks, deparaffinized and rehydrated in a graded ethanol series (100, 90, 70, and 30%), and transferred to PBS solution (137 mM NaCl, 2.7 mM KCl, 4.3 mM Na_2_HPO_4_, 1.4 mM KH_2_PO_4_) for 10 min. Tissues were treated with DNase solution (1 μg/ml) for 10 min at 37°C and washed three times in PBS. Endogenous peroxidase inactivation was carried out by incubating the samples in hydrogen peroxide in methanol for 3% hydrogen peroxide (H_2_O_2_) for 40 min. Sense and antisense probes for CTSF, MMP11 and 12 genes were generated by single-strand PCR using specific cDNA obtained from SiHa cells RNA as template and labeled with biotin-16-dUTP (Roche). Each tissue was covered with 50 μl hybridization cocktail and a coverslip. The hybridization cocktail consisted of 50% formamide, 10% dextran sulfate, 2X SSC (20X SSC: 3 M NaCl, 300 mM Na_3_C_6_H_5_O_7_), PBS, 2% SDS, 100 μg/ml sonicated salmon sperm and 50 ng of dUTP-biotin-labeled probe (sense or antisense). Both the tissue RNA and probe were denatured at 65°C for 10 min. After hybridization, the coverslips were soaked off in Tris-buffered saline with Tween buffer 1X (TBST 10X: 500 nmol/L TrisHCL, pH 7.6, 3 mol/L NaCl, 1% Tween 20), and the tissues were incubated at 55°C for 20 min in stringent wash solution. The horseradish peroxidase labelled antibody (SA-HRP) (GenPoint System from DAKO) was immediately applied on the tissues and incubated for 15 min in a humidifier chamber. The sections were washed in TBST 1X. Biotinyl tyramide was applied to the tissue sections for signal amplification for 15 min at room temperature, and washed in TBST. A second SA-HRP step was carried out and the color reaction was developed with 0.06% diaminobenzidine (DAB) in 3% H_2_O_2_. Finally, the slides were washed, haematoxylin/counterstained, dehydrated in graded ethanol and mounted. Negative controls for ISH were carried out with the sense probes or with a treatment with RNAse solution (100 mg/ml for 30 min at 37°C) prior ISH. Cells were scored as positive for MMP11, MMP12 and CTSF when they showed cytoplasmic expression under the light microscope. Only the neoplastic region of each tissue section was evaluated. To asses the stained cytoplasm, the slides were viewed at 40× magnification. The positivity of cells in each tissue section was estimated by the mean signal intensity, where: (0) positive staining present in at least half of the studied tissue, (1) positive staining of half of the tumor area, and (2) when all tissue had positive staining. The slides were evaluated blindly by three independent observers.

### Immunohistochemical assays (IHC)

Briefly, the tissue sections were deparaffinized and rehydrated in a graded ethanol series. Endogenous peroxidase inactivation was carried out as described in ISH, and the slides were preincubated with DAKO Protein Block Serum free medium Cat X0909 (DAKO, Carpinteria, CA) for 30 min at 37°C to prevent non specific immunoreaction. Excess medium was decanted ant tissues were incubated with the primary antibody as follows: MMP 11 (Biomeda, Cat V10221, Lot 10441, 1:100), MMP12 (R&D Systems, Cat MAB917, Lot AGEO22051. 1:100) at 37°C for 30 min, after which, DAKO Envision System Peroxidase (DAKO) was applied and the slides were counterstained with hematoxylin. As for the *In situ *hybridization, only cells in the neoplastic region of each tumor were evaluated and only when staining for the two proteins were present in the cytoplasm. A rough scoring was done to quantitate the intensity of the staining by three authors (GVO, MSV and PPS). Levels of MMP11 and MMP12 expression in tissue sections were scored under the light microscope. Scores were obtained by estimating mean signal intensity (scale 0 to 2). Scoring was achieved as for ISH. After the examination of the slides by the three independent observers, a global agreement regarding the results was reached.

## Results

### Changes in cDNA expression in CC

Initial analysis of the three normal cervical tissues by limma and marray package showed that the expression profiles were similar. Thus, the data of the histologically normal tissue samples were pooled to generate a mean normal tissue array. Data from the cell lines and tissue arrays were compared with the mean normal tissue arrays. The frequency of increased or decreased gene expression changes was determined by comparing the average normal tissue array with each of the cell lines and tissue arrays. The cut-off values were 4 fold over the normal for up-regulated, and -1 fold for down-regulated or suppressed genes. Fourteenl genes were overexpressed (*TIMP1, Integrins alpha 1 and 4, cadherin 2 and 11, Cathepsins F, B L2, MMP 9, 10 11 and 12*), and 10 genes were down-regulated (*Cathepsin S, L, H and C, Cadherins 3 and 4, TIMP3, MMP 13, Elastase 2 and Integrin beta 8*, Table 1).

### Verification of cDNA expression array results by RT-PCR

**Figure 1:**
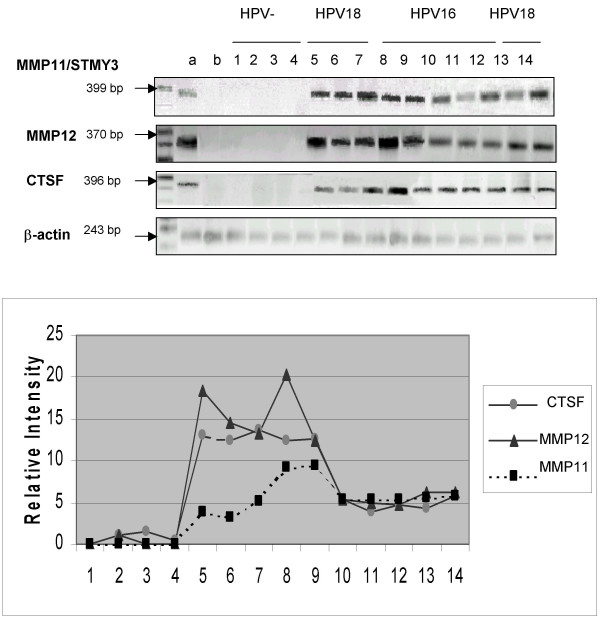
Relative mRNA expression levels of CTSF, MMP11 and MMP12 in cervical tumors detected by RT-PCR. One hundred of total RNA was employed for RT-PCR experiments. The used samples were; line a) positive control, line b) negative control, line 1) normal HPV- tissue #N07, 2) normal HPV- tissue #N11, 3) normal HPV- tissue #N20, 4) normal HPV- tissue #N23, 5) HeLa, 6) CaLO, 7) INBL, 8) CaSki, 9) SiHa, 10) tumoral sample #T04, 11) tumoral sample #T24, 12) tumoral sample #T43, 13) tumoral sample #T45, 14) Tumoral sample T63. A 100 bp ladder was used as molecular weight marker. Values in the graph are presented as ratio of densitometric readings of samples to corresponding beta actin samples.

The gene expression profile findings in cDNA arrays were confirmed by endpoint RT-PCR with densitometry for selected genes (*CTSF*, *MMP11*, *MMP12*, and β-*actin*; Fig. 1). In general, these analyses showed significant results consistent with those obtained by cDNA arrays. All 8 samples investigated by means of cDNA arrays showed increased *CTSF *expression, and in line with this, the tumor band intensities in agarose gels, as compared with the control band intensity, were increased from an average of 1.2 to 7 fold in all tumors for *CTSF *in the RT-PCR analyses. All samples studied by cDNA microarray analysis had increased *MMP11 *expression, and had a mean of 6 fold increased expression by RT-PCR. The greatest difference between the two methods was found in *MMP12 *expression. All samples studied showed increased *MMP12 *expression by cDNA array analysis and RT-PCR. All comparisons were statistically significant.

**Figure 2:**
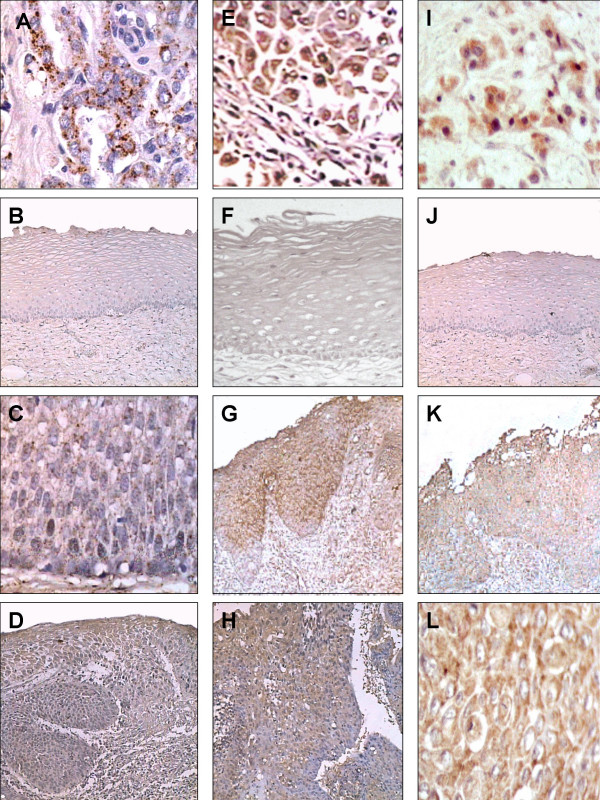
*In situ *hybridization of cervical tissue microarrays with CTSF, MMP11 and MMP12 probes. A) Breast cancer (as positive control of CTSF), B) normal cervical epithelium negative for CTSF, C) HSIL with positive immunostaining, D) CC tissue positive for CTSF hybridization, E) Breast cancer (as positive control for MMP11), F) normal cervical epithelium, negative for MMP11 hybridization, G) as in C, HSIL positive for MMP11 hybridization, H) positive hybridization for MMP11 in CC, I) Breast cancer as positive control for MMP12, J) MMP12 negative hybridization in normal epithelium, K) as in C and G HSIL positive for MMP12, and L) Positive hybridization for MMP12 in CC. Original magnifications were 40 X for A, C, E, I and L; and 10X were used for B, D, F, G, H, J, and K.

ISH was performed on HSIL and CC cases. A strong focal signal (+++) was observed in all CC for the three genes with 90% and 100% of positive. The number of positive cells present in CC (+++) was higher than in HSIL (++). CTSF was expressed in 90%, MMP11 in 90%, and MMP12 in 90% of HSIL studied samples in the tissue array. ALL HSIL showed numerous positive cells for the three transcripts studied. (Fig. 2C, G and 2K; Table 2). In most HSIL MMP11, MMP12 and CTS staining was seen exclusively in the epithelium. The number of positive cells in HSIL was higher than in LSIL (++, +). All three genes were stained in 90% of LSIL (+) samples, while the signal was weaker or undetectable in histological normal cervical tissues (Table 2, Fig. 2). H&E staining at higher magnification in an adjacent tissue slice revealed that the expression of CTS, MMP11 and MMP12 was usually confined to the cytoplasm of a subset of epithelial tumor cells with morphological features of squamous differentiation. Almost all samples showed heterogeneous staining along the tumor, with a few cases showing a stronger reaction in basal layer cells. No staining was observed in the adjacent stroma.

**Figure 3:**
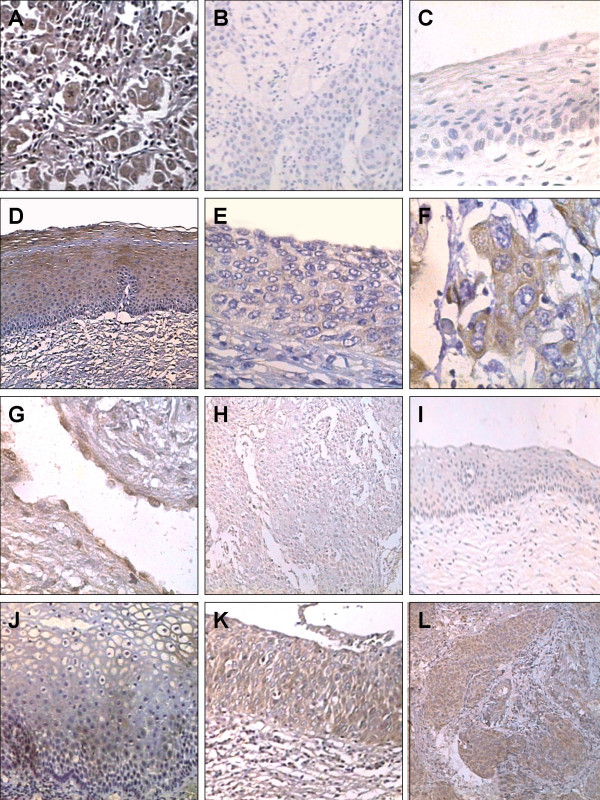
Immunohistochemical detection of MMP11 and MMP12 by cervical tissue microarrays. A) Breast cancer sample (as positive control for MMP11 staining), B) negative control; a CC sample without primary antibody, C) one normal cervical tissue showing negative staining for MMP11, D) LSIL which presents positive immunostaining for MMP11, E) HSIL positive staining for MMP11 F) a CC showing positive immunoreaction for MMP11, G) Positive control for MMP12, H) as in B, negative control for MMP12, I) normal cervical tissue shows negative immunostaining for MMP12, J) LSIL showing positive immunoreaction for MMP12, K) HSIL showing immunodetection for MMP12 and L) a CC showing positive immunoreaction for MMP12. Original magnifications were 40 X for A, C, D, E, F, G, J, and K; and 10X were used for B, H, I, and L.

By immunostaining, the MMP11 and MMP12 proteins were present only in the cytoplasm. There was a strong correlation between ISH and IHC positive reactions. All CC samples had a large number of positive cells (+++, ++) for both proteins, the number of positive cells was lower in HSIL (+++, ++). As seen with ISH, LSIL staining was lower than in HSIL (+) and none of the normal tissues stained for any of the studied proteins (Table 2, Figure 3). Immnunostaining was heterogeneous in CC and in HSIL, whereas in LSIL the staining was preferentially seen on the basal epithelial cells. No staining was observed in the adjacent stroma.

## Discussion

To identify novel genes that could be associated with the development CC, we screened a cDNA-based gene expression array of more than 8000 genes. From those genes found to be significantly upregulated by gene expression analysis, we initially focused on 24 because of their possible involvement in invasiveness and metastasis, 14 of these genes were found to be expressed only in tumor tissue. The present report constitutes the detailed analysis of three such genes: MMP11 MMP12 and cathepsin F. Overexpression of CTSF, MMP11 and MMP12 in CC was confirmed by means of end point RT-PCR. All transcripts were detected in CC, suggesting that these genes are essentially active in the invasive processes of cervical cancer. It is important to note that by ISH these genes were found to be progressively expressed through different stages of cervical carcinogenesis, except for two cases of HSIL, one probable reason of this could be: a possible regression of the disease.

Many normal biological processes, including reproduction, fetal development and wound healing, are critically dependent on controlled degradation of extracellular matrix (ECM). However, excessive degradation of matrix components also occurs in pathologic tissue destruction such as cancer [32]. MMP11 expression in the immediate vicinity or within cancer cells has been associated with some human carcinomas and it is a consistently active partner of invading cancer cells. Its function differs throughout cancer progression, where in can be a tumor enhancer or a repressor in a number of processes leading to local or distal invasion and promoting cancer survival in the stromal environment [33]. We found expression of MMP11 in cervical precancerous lesions but not in histological normal cervix tissues. MMP11 expression was significantly higher in invasive carcinomas. Positive immunoreaction was detected in the cytoplasm of cervix epithelium tumor cells, differing from other tumor tissues, where MMP11 expression is restricted to stromal cells that surround the neoplastic area[34].

MMP11 expression has been reported in pre-invasive bronchial lesions and in carcinomas. MMP11 expression in bronchial lesions starts in dysplasia and carcinoma *in situ*, increasing in invasive lesions, as it appears to occur in CC. Expression of MMP11 in intraepithelial squamous lesions suggest that, as in squamous lung carcinomas, it could be related to progression of phenotypic alterations acquired early during the malignant transformation pathway of cervical epithelium and it is maintained after invasion[35]. It has been previously reported that when MMP11 is increased in tumorigenesis, this is not due to increased neoangiogenesis or cancer cell proliferation, but from a decrease of cancer cell death by apoptosis and necrosis. These data suggest that MMP11 could play different roles trough distinct pathways at different stages of cervical tumor development and progression. This needs to be further studied in CC.

The biological role of MMP12 in tumor progression is not clearly understood but it is thought to be involved in the degradation of components of the basement membrane[36]. Moreover, previous reports have shown that transformed epithelial cells in skin and vulvar cancers express MMP12 and such expression correlates with epithelial dedifferentiation and invasive aggressiveness. In oral verrucous and squamous cell cancer, the absence of MMP12 from epithelial cells has been reported to be a marker of good prognosis in non-invasive oral carcinoma[37].

MMP12 expression in cervical dysplasias and carcinomas has not been previously reported. Expression of MMP12 was detected in most LSIL, HSIL, and all CC samples, but it was absent in normal tissues, probably meaning that this protein is closely involved in early stages of transformation and invasion. By immunohistochemical assays, MMP11 and 12 proteins were detected in LSIL and HSIL with similar patterns as in ISH. Staining was mainly detected in basal cells, probably indicating their degradative and invasive capability.

Although only little data are available on CTSF, analysis of its expression in humans has revealed that it is present in most normal tissues, suggesting a general role for this enzyme in lysosomal function in all cell types. However, CTSF expression levels in normal tissues exhibit a wide variability depending on the tissue type. Several human cancer cell lines have increased expression of CTSF compared to its normal counterpart. This suggests that this enzyme could play an important role in carcinogenesis. Our results are in agreement with a previous study which revealed high levels of CTSF in HeLa cells[38], but it has not been described in cervical cancer. Due to a lack of availability of a specific CTSF antibody, we did not perform IHC assays for this protein. However, by means of ISH, CTSF expression pattern resembles that of MMP11 and 12.

Finally, two more genes found to be overexpressed in this study were MMP9 and TIMP1 (tissue inhibitor of metalloproteinase 1, Table 1). Interestingly, MMP9 and MMP2 up-regulation in cervical cancer was previously reported [33-43] and the combined overexpression of MMP9, MMP11 and TIMP1 has been associated with the invasive features of some cancers[44]. These proteins are clearly associated with CC and their overxpression in this work validates the approach employed.

## Conclusion

In conclusion, our results show that CTSF, MMP11 and 12 are expressed in dysplastic and tumoral epithelial cells suggesting all of these proteins could be used as "potential" progression markers for cervical cancer. The understanding of the role, if any, of these proteins in the pathogenesis of CC deserves further studies.

## List of abbreviations

HPV: Human Papilloma Virus, ISH: In Situ Hybridization, IHC: Immunohistochemistry, CTSF: Cathepsin F.

## Competing interests

The author(s) declare that they have no competing interests.

## Authors' contributions

GVO performed all molecular assays, designed the study, data collection and analysis, and drafted the manuscript.

PPS carried out the IHC and in the tissue block collection, participated in drafting the manuscript.

KV and PM were involved in fresh tissue collection and preparation of tissue blocks.

AD, LT, were involved in carrying out the tissue analysis and data acquisition.

JAG was involved in statistical analysis and writing and revising the manuscript critically.

MS conceived and designed the study, coordinated and managed the study, performed data analysis.

All authors read and approved the final manuscript.

## Pre-publication history

The pre-publication history for this paper can be accessed here:



## Figures and Tables

**Table 1:** Summary of altered gene expression in cervical cancer by cDNA array. Ratio (T/N), intensity ratio of the corresponding signals between tumor and normal cervical tissue.

Gene Name	Unigene	Locus	Hela/n	CaLO/n	INBL/n	SiHa/n	CaSki/n	T07/n	T31/n	T64/n
tissue inhibitor of metalloproteinase 1	Hs.446641	Xp11.3	7	4	4	3	4	4	4	4
integrin, alpha 9	Hs.222	3p21.3	7	6	3	4	3	4	4	3
cadherin 2, type 1, N-cadherin	Hs.334131	18q11.2	6	4	4	5	4	5	4	6
cathepsin L2	Hs.87417	9q22.2	9	5	4	4	4	4	4	4
matrix metalloproteinase 12	Hs.1695	11q22.3	5	9	8	9	6	5	4	5
integrin, alpha 4	Hs.145140	2q31	8	3	5	4	3	4	5	4
serine B member 9	Hs.104879	6p25.2	5	4	4	5	4	3	4	4
serine, proteinase inhibitor, clade B member 3	Hs.227948	18q21.3	6	4	4	5	4	4	4	3
cathepsin B	Hs.297939	8p22	6	4	3	5	5	4	4	4
cathepsin F	Hs.11590	11q13	8	8	6	7	6	5	5	5
matrix metalloproteinase 10	Hs.2258	11q22	7	5	5	3	4	4	4	5
matrix metalloproteinase 11 (stromelysin 3)	Hs.143751	22q11.23	6	8	6	6	6	5	5	5
matrix metalloproteinase 9	Hs.151738	20q13.12	5	6	5	4	4	5	4	3
cadherin 11, type 2	Hs.443435	16q22.1	6	3	3	3	4	5	5	4
cathepsin S	Hs.181301	1q21	0	1	1	0	1	1	0	1
cathepsin L	Hs.418123	9q21.33	0	0	1	1	1	0	0	1
cathepsin H	Hs.114931	15q25.1	0	1	0	1	0	0	1	0
cathepsin C	Hs.128065	11q14.2	1	0	1	0	1	1	1	0
matrix metalloproteinase 13	Hs.2936	11q22	0	0	0	1	0	1	0	0
tissue inhibitor of metalloproteinase 3	Hs.245188	22q12.3	0	1	0	1	1	1	1	0
cadherin 3, type 1, P-cadherin (placental)	Hs.191842	16q22.1	0	1	1	0	0	1	0	1
cadherin 4, type 1, R-cadherin (retinal)	Hs.376792	20q13.3	1	0	1	0	1	1	0	1
integrin, beta 8	Hs.355722	7p21.1	0	0	0	1	1	0	0	1
elastase 2, neutrophil	Hs.99863	19p13.3	0	1	0	0	1	0	0	0

**Table 2:** Summary of the *In situ *hybridizations and immunohistochemical stainings results of cervical tissue microarrays.

		**% Positivity in ISH**											
		**CTSF**				**MMP11**				**MMP12**			

	**Total of cases**	**-**	**(+)**	**(++)**	**(+++)**	**-**	**(+)**	**(++)**	**(+++)**	**-**	**(+)**	**(++)**	**(+++)**

Normal tissues	15	14	1	0	0	13	2	0	0	13	2	0	0
LSIL	10	1	9	0	0	1	9	0	0	1	9	0	0
HSIL	10	1	1	8	0	1	2	7	0	1	2	7	0
CC tissues	15	0	0	0	15	0	0	0	15	0	0	0	15
						**% Positivity in IHC**							

						**MMP11**				**MMP12**			

	**Total of cases**					**-**	**(+)**	**(++)**	**(+++)**	**-**	**(+)**	**(++)**	**(+++)**

Normal tissues	15					15	0	0	0	15	0	0	0
LSIL	10					0	10	0	0	0	9	1	0
HSIL	10					0	2	8	0	0	0	8	0
CC tissues	15					0	0	5	10	0	0	1	14
