# Correction: Overexpression of cathepsin f, matrix metalloproteinases 11 and 12 in cervical cancer

**DOI:** 10.1186/1471-2407-5-164

**Published:** 2005-12-29

**Authors:** Guelaguetza Vàzquez-Ortiz, Patricia Piña-Sanchez, Karla Vázquez, Alfonso Dueñas, Lucia Taja, Patricia Mendoza, José Antonio García, Mauricio Salcedo

**Affiliations:** 1 Oncogenomics Laboratory, Oncology Research Unit, Oncology Hospital, National Medical Center SXXI-IMSS, Mexico; 2 Division of Basic Research, INCAN, SS, Mexico; 3 Laboratory of Theoretical Biology, Research Department, La Salle University, Mexico

After the publication of our work in BMC Cancer [1], we became aware of an omission. In the Methods section, under the subtitles "Cell lines and tissue samples for cDNA arrays" and "cDNA probe preparation" we failed to quote the original description of how the cDNA library was generated, published in the Journal Archives of Medical Research as quoted below [2]. Since this initial analysis, further data mining has been performed, and the obtained results answer very different biological questions [1].

We regret any inconvenience that this involuntary inaccuracy in the original data citation might have caused. We wish to thank Dr. Fabio Salamanca for bringing this matter to our attention.

## Pre-publication history

The pre-publication history for this paper can be accessed here:


